# Secondhand Smoke Exposure in Hospitality Venues in Europe

**DOI:** 10.1289/ehp.11374

**Published:** 2008-07-18

**Authors:** Maria J. Lopez, Manel Nebot, Marco Albertini, Pierre Birkui, Francesc Centrich, Monika Chudzikova, Maria Georgouli, Giuseppe Gorini, Hanns Moshammer, Maurice Mulcahy, Maria Pilali, Eulalia Serrahima, Piotr Tutka, Esteve Fernandez

**Affiliations:** 1 Evaluation and Intervention Methods Unit, Public Health Agency of Barcelona, Barcelona, Spain; 2 PhD Program in Public Health and Methodology of Biomedical Research, Universitat Autònoma de Barcelona, Barcelona, Spain; 3 CIBER Epidemiología y Salud Pública, Spain; 4 Department of Experimental and Health Sciences, Pompeu Fabra University, Barcelona, Spain; 5 Centro Regionale di Riferimento per la Prevenzione, Florence, Italy; 6 Office Français de Prevention du Tabagisme, Paris, France; 7 Laboratory, Agency of Public Health, Barcelona, Spain; 8 Stop Smoking NGO, Bratislava, Slovak Republic; 9 Hellenic Cancer Society, Athens, Greece; 10 Environmental and Occupational Epidemiology Unit, Centre for Study and Prevention of Cancer–Tuscan Cancer Institute, Florence, Italy; 11 Institute of Environmental Health, Medical University of Vienna, Vienna, Austria; 12 Health Service Executive, Western Area, Galway, Ireland; 13 Department of Experimental and Clinical Pharmacology, Medical University of Lublin, Lublin, Poland; 14 Tobacco Control Research Unit, Institut Català d’Oncologia-IDIBELL, Barcelona, Spain; 15 Department of Clinical Sciences, Campus of Bellvitge, Universitat de Barcelona, Barcelona, Spain

**Keywords:** Europe, hospitality sector, passive smoking, secondhand smoke, vapor-phase nicotine

## Abstract

**Background:**

Although in the last few years some European countries have implemented smoking bans in hospitality venues, the levels of secondhand smoke (SHS) in this occupational sector could still be extremely high in most countries.

**Objective:**

The aim of this study was to assess exposure to SHS in hospitality venues in 10 European cities.

**Methods:**

We included 167 hospitality venues (58 discotheques and pubs, 82 restaurants and cafeterias, and 27 fast-food restaurants) in this cross-sectional study. We carried out fieldwork in 10 European cities: Vienna (Austria), Paris (France), Athens (Greece), Florence and Belluno (Italy), Galway (Ireland), Barcelona (Spain), Warsaw and Lublin (Poland), and Bratislava (Slovak Republic). We measured vapor-phase nicotine as an SHS marker.

**Results:**

We analyzed 504 samples and found nicotine in most samples (97.4%). We found the highest median concentrations in discos/pubs [32.99 μg/m^3^; interquartile range (IQR), 8.06–66.84 μg/m^3^] and lower median concentrations in restaurants/cafeterias (2.09 μg/m^3^; IQR, 0.49–6.73 μg/m^3^) and fast-food restaurants (0.31 μg/m^3^; IQR, 0.11–1.30 μg/m^3^) (*p* < 0.05). We found differences of exposure between countries that may be related to their smoking regulations. Where we sampled smoking and nonsmoking areas, nicotine concentrations were significantly lower in nonsmoking areas.

**Conclusions:**

Hospitality venues from European cities without smoking regulations have very high levels of SHS exposure. Monitoring of SHS on a regular basis as well as a total smoking ban in hospitality sector would be needed.

Involuntary exposure to secondhand smoke (SHS) causes premature death and disease, including cancer and cardiovascular and respiratory diseases [Centers for Disease Control and Prevention (CDC) 2006]. Hospitality workers (i.e., workers in bars, pubs, restaurants, and other venues) are exposed to much higher levels of SHS compared with other workers (Hahn et al. 2006; Siegel and Skeer 2003; Wakefield et al. 2005). A review including 13 studies conducted in the United States that measured SHS exposure among hospitality workers concluded that the excess lung cancer mortality risk would be 410 deaths per 100,000 workers exposed (Siegel and Skeer 2003), a risk that could be even higher in countries with higher levels of exposure (Lopez et al. 2006). Different studies have also shown higher prevalence of respiratory symptoms among hospitality workers that decreased significantly in those countries that implemented smoke-free policies in the hospitality sector (Allwright et al. 2005).

In the last few years, some European countries, including Ireland and Italy, have implemented complete smoke-free policies in workplaces, including hospitality venues (Allwright et al. 2005; Gorini et al. 2005; Haw et al. 2006; Howell 2004; Joossens and Raw 2006). Smoking bans in other European countries have generally excluded hospitality venues. As a result, the level of exposure to SHS in this occupational sector is still very high in most European countries. To objectively document levels of exposure to SHS in hospitality venues across Europe and to compare the exposure among these cities regulated with different policies, we measured airborne nicotine concentrations in hospitality venues in 10 European cities using a common protocol.

## Materials and Methods

### Design and population

This multicountry cross-sectional study is part of a study funded by the European Commission that aimed to measure SHS exposure in three occupational sectors. We carried out the fieldwork from March 2004 to March 2005 in major cities from eight European countries: Vienna (Austria), Paris (France), Athens (Greece), Florence and Belluno (Italy), Galway (Ireland), Barcelona (Spain), Lublin and Warsaw (Poland), and Bratislava (Slovak Republic). A common protocol was discussed and agreed by all the partners in a preparatory meeting.

We grouped hospitality venues in the study in three categories: discotheques and pubs, restaurants and cafeterias, and fast-food restaurants. We defined discos and pubs as any kind of musical bar open at night, restaurants and cafeterias as hospitality venues where food and drinks are served, and fast-food restaurants as quick-service restaurants characterized by fast-food cuisine and minimal table service. We selected the venues following a convenience sampling based on the type of setting, geographic area, and smoking regulation. Within each establishment, we placed the nicotine samplers in areas commonly occupied by workers and patrons. For establishments with smoking restrictions, we placed samplers in smoking and nonsmoking areas. The total number of samplers per establishment ranged from one to four (one or two samplers per venue, except for places with smoking and nonsmoking sections, where we placed one or two samplers in each section). Table 1 shows the number of establishments and final number of nicotine measurements by country and venue category, as well as country data on smoking prevalence and smoking regulation in hospitality venues at the time of the study.

### Nicotine measurements

We measured vapor-phase nicotine using SHS passive samplers, following the method described and validated by Hammond (1993). In summary, the samplers consist of a 37-mm-diameter plastic cassette containing a filter treated with sodium bisulfate. We placed the samplers for 7 days in cafeterias, traditional restaurants, and fast-food restaurants. The instructions to place the SHS samplers were as follows: Samplers had to hang freely in the air and were not to be placed where air does not circulate (e.g., a corner, under a shelf, or buried in curtains). In discos and pubs, samplers were carried by a person during 4–5 hr. For each sample, we recorded the following data: Sample’s code, country, public place, sample location, and date and time placed and removed. We recorded information on sampling area, sampling volume, and ventilation in each establishment for evaluation of extreme or inconsistent values. The nicotine analysis was conducted at the Laboratory of the Public Health Agency of Barcelona by gas chromatography/mass spectrometry. The lower limit of detection was 0.01 μg/mL. We estimated the time-weighted average nicotine concentration (micrograms per cubic meter) by dividing the amount of extracted nicotine by the volume of air sampled [estimated flow rate (24 mL/min) times total number of minutes the filter had been exposed].

### Statistical analysis

We used medians and interquartile ranges (IQRs) to describe the data by country for each hospitality category. We compared medians using the nonparametric comparison test of medians (Sheskin 1997). We performed analyses using SPSS 13.0 (SPSS Inc., Chicago, IL, USA).

## Results

We analyzed 504 samples placed in 167 establishments in the eight participating countries (Table 1). We detected airborne nicotine in most samples (97.4%). As shown in Table 2, we found the highest concentrations in discos/pubs, whereas in restaurants and cafeterias and fast-food restaurants the nicotine concentrations were lower (*p* < 0.05).

We also found differences by city (*p* < 0.05). In discos/pubs, we found the highest nicotine concentration in Barcelona and the lowest in Galway. In restaurants and cafeterias, nicotine concentrations were highest in Bratislava and Vienna and lowest in Galway. In fast-food restaurants, nicotine concentrations were highest in Barcelona, followed by Vienna, and lower in the rest of countries (median concentrations < 1 μg/m^3^).

In venues with smoking and nonsmoking areas (see Table 3), the median nicotine concentration is significantly higher in smoking areas than in nonsmoking areas. The ratio of smoking areas to nonsmoking areas was 3.12, with a median concentration in smoking areas and nonsmoking areas of 4.40 μg/m^3^ and 1.41 μg/m^3^, respectively (*p* < 0.05).

## Discussion

We found SHS exposure in most places studied, with differences of exposure between countries that may be related with their smoking regulations. Exposure to SHS was high or very high in most hospitality venues across the European countries evaluated in this study, except Ireland. At the time of the study, only Ireland had complete smoke-free regulations covering the hospitality sector, suggesting that complete smoke-free regulations can effectively protect workers from occupational exposure to SHS. This study provides for the first time objective data of SHS levels in an important sample of hospitality venues from three hospitality sectors of eight European countries. Despite the differences across countries, there is a general pattern of exposure by hospitality setting, with fast-food restaurants being the places with the lowest levels of SHS and discos and pubs the settings with the highest levels. In venues with separated areas, the median nicotine concentration in the smoking areas was more than three times as high as the concentration in the nonsmoking areas, where the median concentration was 1.41 μg/m^3^.

Comparing our results with those obtained 2 years before by Nebot et al. (2005) seems to show a general trend of decrease in the nicotine concentrations. The median nicotine concentration decreased in discos and pubs in Paris (58.91–32.64 μg/m^3^), Athens (98.09–30.80 μg/m^3^), and Vienna (122.24–30.38 μg/m^3^), whereas in Barcelona there are no significant differences (from 91.44–113.78 μg/m^3^). In restaurants, the median levels of nicotine concentration in Paris (6.06–1.18 μg/m^3^), Athens (4.70–3.99 μg/m^3^), Vienna (17.74–9.94 μg/m^3^), and Barcelona (7.81–3.62 μg/m^3^) are also lower than in the previous study.

When comparing with other geographic areas (Table 4), the nicotine concentrations found in restaurants (2.09 μg/m^3^) are higher than in Latin America (1.24 μg/m^3^) and quite similar to the levels found in China (2.17 μg/m^3^). The results obtained in discos and pubs in our study (32.99 μg/m^3^) are consistent with the results found by Siegel and Skeer (2003) in bars in the United States (~ 31.1 μg/m^3^). However, the results found in bars from Latin America were nearly 10 times lower (3.65 μg/m^3^) than those found in our study. This could be explained by the warm weather in Latin America, which may result in more ventilated venues.

Other studies have quantified the SHS levels in hospitality sector using biomarkers such as nicotine in hair or cotinine in saliva, or using airborne markers, all them concluding that SHS exposure levels in hospitality sector are specially high (Hahn et al. 2006; Wakefield et al. 2005). It is especially important to highlight that this study allows the comparison of levels of SHS exposure between cities with smoking regulations in the hospitality sector and those that do not have any smoking regulation in these settings. The data obtained show the benefits of smoking prohibitions on the control of the SHS exposure. In Ireland, where we found the lowest values, we carried out the fieldwork after the implementation of the Irish anti-smoking law (March 2004). Some Irish studies (Allwright et al. 2005; Mulcahy et al. 2005) evaluating the impact of the Irish law showed significant decreases in the levels of SHS. Allwright et al. (2005) found a decrease of 80% of saliva cotinine in hospitality workers, and Mulcahy et al. (2005) found a similar decrease (83%) in air nicotine concentrations, with a postlaw concentration of 5.9 μg/m^3^ in bars. In Italy, we took half of the nicotine measurements of our study in discos and pubs before the implementation of their law (January 2005), and the other half after implementation. The results, already published by Gorini et al. (2005), show an impressive decrease in the levels of SHS.

The method of nicotine measurement has been widely used and validated in numerous studies (Hammond 1993; Lopez et al. 2004; Navas-Acien et al. 2004; Nebot et al. 2005). It is a sensitive and specific indicator for SHS (CDC 2006) that has been used to evaluate smoking laws in several countries, such as Italy and Ireland (Allwright et al. 2005; Mulcahy et al. 2005). This marker has been also used in multicountry studies in Europe, Latin America, and China (Navas-Acien et al. 2004; Nebot et al. 2005; Stillman et al. 2007).

However, some limitations regarding the sampling should be taken into account. Because we sampled the venues that agreed to participate in a convenience basis, we cannot exclude some underestimation of the SHS levels (e.g., owners of venues with relatively low levels may be more likely to permit sampling), but this would be a conservative bias. Nevertheless, the data observed in our study are not lower but are consistent with other studies (Siegel and Skeer 2003; Stillman et al. 2007). We did the sampling in a convenience basis, because the main goal of the project was not to have a representative sample of hospitality venues but to have sufficient data on SHS exposure in Europe in a wide range of hospitality settings, using an objective marker and minimizing the absence of information bias. Furthermore, we followed a common protocol in all the countries to standardize the methodology and to strengthen the comparability of results. In pubs and discos, samplers were exposed for shorter periods (4–5 hr) than in other settings, so these results are not directly comparable with those obtained in the other settings. However, we did this because these venues have most of their clients on weekends, and some of them are only open at this time. Therefore, exposing a sampler for a whole week would have underestimated the real exposure. In addition, because nicotine concentrations in these settings during working hours are very high, a minimum of 4 hr is sufficient to detect the presence of nicotine in these venues. Finally, except for discos and pubs, where we used samplers a few hours, we assessed nicotine concentrations dividing the total amount of nicotine in the filter by the total exposure, including nights, when there are neither people smoking nor people exposed. For this reason, these data could be underestimating the real exposure of workers, who are working during time periods when the concentration is higher. However, we considered it more suitable to use the most conservative assumptions.

The median nicotine concentration found in the venues of our study is associated with an excess lung cancer mortality risk of 438 per 100,000 in discos and 28 per 100,000 in restaurants, assuming a regular exposure (8 hr per working day) to these levels of SHS during a 40-year working period (derived using the formula of Repace and Lowrey 1993). Hospitality workers may represent a more transient occupational group, but the assessment of the level of occupational risk for these workers should be based on whether it would be safe for them to work under such conditions for a working lifetime. Furthermore, we estimated only the excess lung cancer deaths, but the number of heart disease deaths attributable to SHS exposure could far exceed the number of lung cancer deaths.

In summary, hospitality workers in Europe, especially those working in discos and pubs, are occupationally exposed to very high levels of SHS. Some European countries, however, have excluded hospitality venues from smoking regulations (Fernandez 2006). These high concentrations of exposure to SHS cause serious health risks for workers in the hospitality sector, including lung cancer and cardiovascular and respiratory diseases, and represent fundamental inequalities in working conditions and occupational safety standards. To protect workers from the health consequences of SHS, complete smoke-free regulations, including the hospitality sector, are urgently needed in all European countries. Future research should monitor SHS exposure on a regular basis and evaluate the long-term success of smoke-free regulations in protecting hospitality workers from SHS exposure.

## Figures and Tables

**Table 1:** Description of relevant information about smoking prevalences in adults, smoking regulations in the hospitality sector, and fieldwork information in the countries studied.

	Country information		Fieldwork information: no. of samples and settings studied							
	Smoking prevalence [% (year)]	National smoking regulations^a^	Disco/pubs		Restaurants/cafeterias		Fast-food restaurants		Total	
Country			Samples	Establishments	Samples	Establishments	Samples	Establishments	Samples	Establishments
Austria	29^b^ (2000)	Not regulated	23	7	23	9	11	4	57	20
Greece	37.6^b^ (2000)	Not regulated	17	5	20	4	8	2	45	11
France	27^b^ (2000)	Restricted	17	7	24	8	11	4	52	19
Ireland	31^b^ (1998)	Banned	10	3	36	11	6	2	52	16
Italy	31.1 (males); 22.3 (females)^b^ (2002)	Not regulated or restricted (new law from January 2005)	41	12	35	8	8	3	84	23
Poland	34^b^ (1997–1999)	Restricted	30	10	45	15	9	2	84	27
Slovak Republic	32^b^ (1998)	Restricted	24	7	36	12	18	6	78	25
Spain	31^c^ (2003)	Not regulated	13	7	29	15	10	4	52	26
Total			175	58	248	82	81	27	504	167

a March 2004–March 2005. Categories are as follows: Not regulated: no smoking regulation exists affecting hospitality sector; Restricted: smoking in hospitality sector is not totally banned, but there are some restrictions; Banned: smoking is prohibited in all hospitality venues.

b Data from Shafey et al. (2003).

c Data from Ministerio de Sanidad y Consumo (2007).

**Table 2:** Median (IQR) nicotine concentration (μg/m^3^) by country and type of hospitality venue.^a^

Country	Disco/pubs	Restaurants/cafeterias	Fast food
Austria (Vienna)	30.38 (21.70–74.40)	9.94 (2.30–21.66)	1.10 (0.17–3.24)
Greece (Athens)	30.80 (23.01–60.33)	3.99 (2.00–6.38)	0.74 (0.64–0.91)
France (Paris)	32.64 (1.17–123.07)	1.18 (0.19–4.84)	0.12 (0.04–0.27)
Ireland (Galway)	6.93 (2.77–11.36)	0.19 (0.14–0.40)	0.15 (0.00–0.25)
Italy (Florence and Belluno)			
Pre-law	138.93 (93.96–207.46)	1.75 (1.20–3.61)	0.97 (0.48–23.68)
Post-law	4.52 (1.74–7.59)	—	—
Poland (Warsaw and Lublin)	18.67 (5.86–65.97)	1.53 (0.23–2.85)	0.13 (0.09–0.32)
Slovak Republic (Bratislava)	44.37 (11.28–57.21)	10.95 (6.22–17.67)	0.07 (0.03–0.27)
Spain (Barcelona)	113.78 (63.46– 239.59)	3.62 (1.02–7.45)	3.76 (1.33–6.06)
Total	32.99 (8.06–66.84)	2.09 (0.49–6.73)	0.31 (0.11–1.30)

a We placed the samplers for 7 days in all settings except for discos/pubs, where the samplers were carried by a person during 4–5 hr.

**Table 3:** Nicotine concentration (μg/m^3^) in smoking and nonsmoking areas by venue [mean (no.)].^a^

Country/venue	Smoking areas	Nonsmoking areas	Ratio of smoking areas to nonsmoking areas
Austria (Vienna)			
Venue 1	1.48 (2)	0.58 (1)	2.55
Venue 2	12.42 (2)	1.61 (1)	7.71
Venue 3	81.25 (2)	6.24 (1)	13.02
Venue 4	15.8 (2)	8.71 (1)	1.81
Poland (Warsaw and Lublin)			
Venue 5	8.68 (2)	2.48 (1)	3.50
Venue 6	2.49 (2)	0.45 (1)	5.53
Venue 7	2.82 (2)	0.52 (1)	5.42
Venue 8	1.35 (2)	1.41 (1)	0.96
Venue 9	3.84 (1)	1.83 (2)	2.10
Venue 10	2.63 (2)	1.43 (1)	1.84
Venue 11	5.17 (1)	1.68 (2)	3.08
Spain (Barcelona)			
Venue 12	17.33 (2)	1.31 (1)	13.23
Venue 13	2.63 (1)	1.34 (1)	1.96
Venue 14	5.90 (2)	4.01 (1)	1.47
Venue 15	3.51 (1)	1.1 (1)	3.19
Total (median)	4.40	1.41	3.12

a Data for smoking and nonsmoking areas are available for only three countries involved in the study.

**Table 4:** Comparison of nicotine concentrations (μg/m^3^) in hospitality venues among major studies.

Study	Geographic area (year of fieldwork)	Discos/pubs/bars [median (IQR) (no.)]	Restaurants [median (IQR) (no.)]
Present study	Europe (2004–2005)	32.99 (8.06–66.84) (175)	2.09 (0.49– 6.73) (248)
Nebot et al. (2005)	Europe (2002–2003)	88.13 (30.54–184.21) (40)	3.58 (0.94–10.05) (100)
Navas-Acien et al. (2004)	Latin America (2002–2003)	3.65 (1.55–5.12) (97)	1.24 (0.41– 2.48) (44)
Siegel and Skeer (2003)	USA (review of different studies)	31.1 (7.4–105.4)^a^ (27)	6.5 (3.4–34.0)^a^ (402)
Stillman et al. (2007)	China (2005)	—	2.17 (1.02–4.63) (54)

a Mean (range).
